# Comparison between genetic and pharmaceutical disruption of Ldlr expression for the development of atherosclerosis

**DOI:** 10.1016/j.jlr.2022.100174

**Published:** 2022-01-29

**Authors:** Diego Gomes, Shari Wang, Leela Goodspeed, Katherine E. Turk, Tomasz Wietecha, Yongjun Liu, Karin E. Bornfeldt, Kevin D. O'Brien, Alan Chait, Laura J. den Hartigh

**Affiliations:** 1 Division of Metabolism, Endocrinology, and Nutrition, Department of Medicine, University of Washington, Seattle, WA, USA; 2 Diabetes Institute, University of Washington, Seattle, WA, USA; 3 Department of Laboratory Medicine and Pathology, University of Washington, Seattle, WA, USA; 4 Department of Pathology and Laboratory Medicine, University of Wisconsin, Madison, WI, USA; 5 Division of Cardiology, Department of Medicine, University of Washington, Seattle, WA, USA

**Keywords:** animal models, hyperlipidemia, inflammation, liver, receptors/lipoprotein, antisense oligonucleotides, hepatic inflammation, serum amyloid A, fast-phase liquid chromatography, ASO, antisense oligonucleotide, *Cpt1a*, carnitine palmitoyl transferase 1 alpha, *Dgat2*, diacylglycerol O-acyltransferase 2, FPLC, fast-phase liquid chromatography, HFHS, high-fat and high-sucrose diet, *Hmgcr*, 3-hydroxy-3-methylglutaryl-CoA reductase, Il6, interleukin-6, LDLR, LDL receptor, *Lgals3*, galectin-3, NIH, National Institutes of Health, SAA, serum amyloid A, SMA, smooth muscle actin, *Srebp1c*, sterol regulatory element-binding transcription factor 1

## Abstract

Antisense oligonucleotides (ASOs) against Ldl receptor (Ldlr-ASO) represent a promising strategy to promote hypercholesterolemic atherosclerosis in animal models without the need for complex breeding strategies. Here, we sought to characterize and contrast atherosclerosis in mice given Ldlr-ASO with those bearing genetic Ldlr deficiency. To promote atherosclerosis, male and female C57Bl6/J mice were either given weekly injections of Ldlr-ASO (5 mg/kg once per week) or genetically deficient in Ldlr (*Ldlr*^−/−^). Mice consumed either standard rodent chow or a diet high in saturated fat and sucrose with 0.15% added cholesterol for 16 weeks. While both models of Ldlr deficiency promoted hypercholesterolemia, *Ldlr*^−/−^ mice exhibited nearly 2-fold higher cholesterol levels than Ldlr-ASO mice, reflected by increased VLDL and LDL levels. Consistent with this, the en face atherosclerotic lesion area was 3-fold and 3.6-fold greater in male and female mice with genetic Ldlr deficiency, respectively, as compared with the modest atherosclerosis observed following Ldlr-ASO treatment. Aortic sinus lesion sizes, fibrosis, smooth muscle actin, and necrotic core areas were also larger in *Ldlr*^−/−^ mice, suggesting a more advanced phenotype. Despite a more modest effect on hypercholesterolemia, Ldlr-ASO induced greater hepatic inflammatory gene expression, macrophage accumulation, and histological lobular inflammation than was observed in *Ldlr*^−/−^ mice. We conclude Ldlr-ASO is a promising tool for the generation of complex rodent models with which to study atherosclerosis but does not promote comparable levels of hypercholesterolemia or atherosclerosis as *Ldlr*^−/−^ mice and increases hepatic inflammation. Thus, genetic Ldlr deficiency may be a superior model, depending on the proposed use.

Over the past several decades, the advent of gene editing technology, particularly in mice, has enabled considerable advancement of our understanding of metabolic disease. In particular, the generation of Ldl receptor (Ldlr)-deficient and Apoe-deficient mice (1, 2) has led to an explosion of atherosclerosis research. As enlightening as they have been, such genetically perturbed models require rigorous breeding strategies in order to study the atherogenicity of complex systems. By contrast, antisense oligonucleotides (ASOs) represent a relatively novel technique that might both alleviate the need for complex breeding strategies and offer the potential for reversibility models. ASOs are small single-stranded molecules, typically less than 50 nucleotides, that specifically bind to a complementary sequence in the targeted mRNA strand. Upon binding, the complex is recognized and degraded by RNase-H cleavage (3), an endogenous cellular mechanism that prevents subsequent protein translation (4), thus inhibiting functional protein expression. Accordingly, ASOs represent a novel tool for the disruption of protein synthesis in a highly specific manner. ASOs have been shown to exhibit efficacy when given pharmacologically to humans in several clinical trials, including the reduction of LDL cholesterol by Apob100-ASO (5), the reduction of plasma triglycerides by Apoc3-ASO (6) and Angptl3-ASO (7), and reduced Lp(a) levels with Apo(a)-ASO (8). However, some Food and Drug Administration-approved ASO medications have been removed from the market because of adverse side effects (i.e., the Apob100-ASO, mipomersan, in 2019). Thus, while ASOs represent a promising platform with the potential to selectively target particular mRNAs in a cell, thereby dramatically expanding potentially druggable disease targets, more work is needed to ensure their safety for use in humans (3).

Many ASOs have been developed because of their potential not only as pharmaceutical agents but also as research tools to study metabolic disease. The Ldlr ASO is one such example, offering researchers the potential to create complex genetic models to study atherosclerosis without the need for time-consuming and challenging breeding strategies. Ldlr-ASO has been a useful tool in this regard, utilized recently to study atherosclerosis regression (9, 10), to enable a reversible atherosclerosis induction model (9), and to examine Ldlr-dependent hepatic HDL flux (11). One of the most well-studied atherosclerotic mouse models, the *Ldlr*^−/−^ mouse, develops atherosclerotic lesions that closely resemble human pathological lesions, including high levels of VLDL and LDL, when fed a diet containing high fat and cholesterol (12, 13). Thus, there is great potential for Ldlr-ASO technology to facilitate the study of atherosclerosis in genetic or temporal mouse models.

In this study, we investigated how Ldlr-ASO would compare with the more commonly used genetic mouse model of Ldlr deficiency. Herein, we compare and contrast the efficacy of Ldlr-ASO and the *Ldlr*^−/−^ mouse to silence the Ldlr, promote hypercholesterolemia, and facilitate atherosclerosis development in both male and female mice. In parallel with *Ldlr*^−/−^ mice consuming a high-fat and high-sucrose (HFHS) diet containing 0.15% cholesterol, mice sufficient in Ldlr (C57Bl6/J mice) received Ldlr-ASO for 16 weeks. We report that while pharmacological and genetic silencing of Ldlr promoted comparable levels of Ldlr deficiency, *Ldlr*^−/−^ mice exhibited augmented plasma cholesterol levels, largely observed in VLDL and LDL fractions. This augmented hypercholesterolemia in *Ldlr*^−/−^ mice resulted in more advanced atherosclerosis in the aorta and aortic sinus when compared with the modest levels observed in Ldlr-ASO-treated mice. Moreover, chronic Ldlr-ASO treatment led to higher levels of hepatic inflammation than in *Ldlr*^−/−^ mice. Thus, while Ldlr-ASO presents a useful tool for use in complex atherosclerosis models, its efficacy at promoting hypercholesterolemia and advanced atherosclerosis are lower than genetic deficiency.

## Materials and methods

### Mouse study design

To promote atherosclerosis, group-housed 10-week-old male and female mice were rendered hypercholesterolemic by one of two methods of *Ldlr* deficiency.

#### Method 1

To pharmacologically silence Ldlr, C57Bl/6J mice (catalog no.: 000664; Jackson Laboratory) were intraperitoneally injected weekly with GalNAc-conjugated Gen 2.5 ASO (5 mg/kg) targeting mouse Ldlr (Ldlr-ASO; catalog no.: ION 713852; Ionis Pharmaceuticals), a scrambled control ASO (cont-ASO; catalog no.: ION 740133; Ionis Pharmaceuticals), or sterile saline for 16 weeks, while consuming either normal chow or a diet high in HFHS. The HFHS diet contains 59% fat and 36% sucrose with 0.15% added cholesterol, as described previously (14). ASO preparations were generous gifts from Ionis Pharmaceuticals (Carlsbad, CA).

#### Method 2

**Fig. 1:**
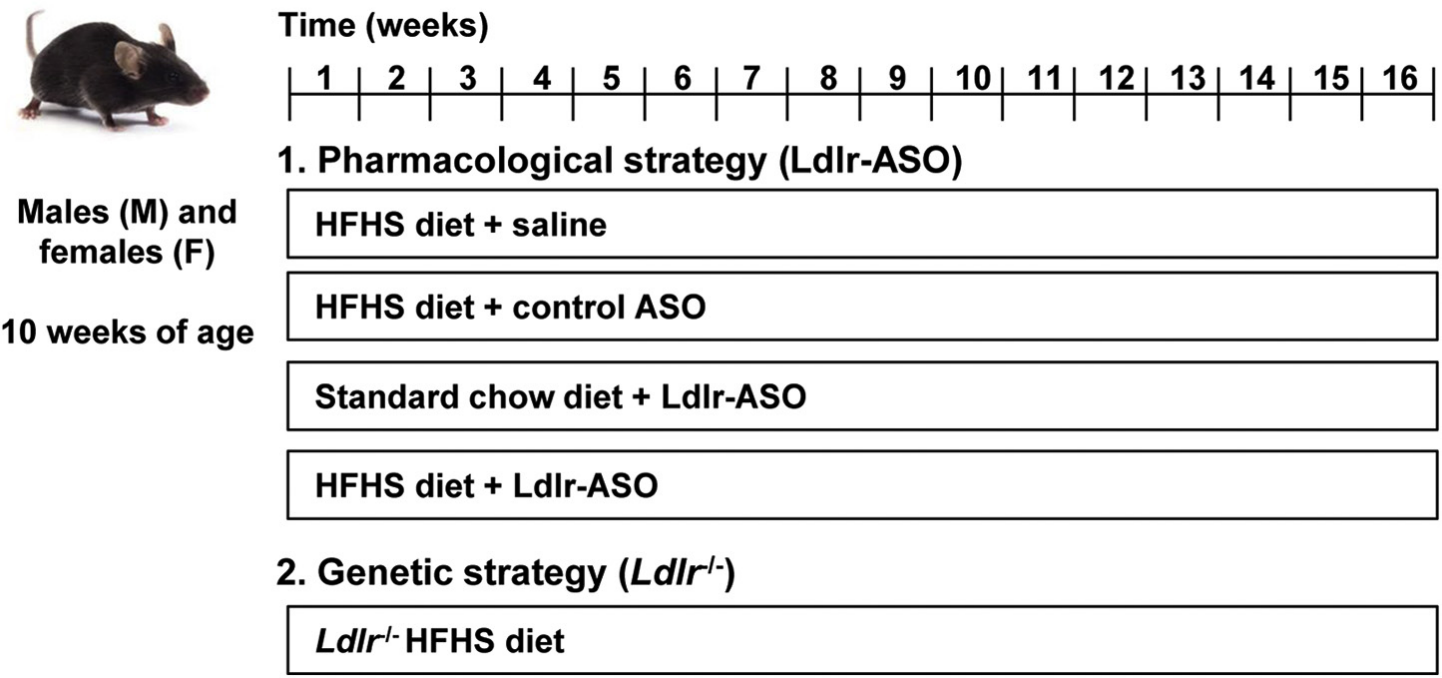
Mouse study design. Two models were utilized to promote atherosclerosis in C57Bl/6J mice. In the pharmacological strategy, male and female mice (10 weeks of age) were fed either a normal chow diet or placed on an HFHS diet for 16 weeks. Mice were given weekly intraperitoneal injections of either saline, control-ASO, or Ldlr-ASO. In the genetic strategy, male and female *Ldlr*^−/−^ mice were fed an HFHS diet for 16 weeks.

Mice genetically deficient in Ldlr (catalog no.: 002207; Jackson Laboratory) began consuming an HFHS diet at 10 weeks of age for 16 weeks. For both methods, blood samples were obtained retro-orbitally every 4 weeks. At sacrifice, blood was collected and PBS-perfused harvested tissues were snap frozen in liquid nitrogen and stored at −80°C or were fixed with 10% neutral-buffered formalin and embedded in paraffin wax. See Fig. 1 for the study design and Table 1 for the number of animals used in each treatment group. All experimental procedures were undertaken with approval from the Institutional Animal Care and Use Committee of the University of Washington and followed the guidelines of the National Institutes of Health (NIH) guide for the care and use of laboratory animals (NIH publications no.: 8023; revised 1978).

**Table 1:** Animal numbers per treatment group

Treatment group	Males	Females
Method 1: Antisense strategy		
HFHS-fed Weekly saline	n = 8	n = 9
HFHS-fed Weekly cont-ASO	n = 4	None
Chow-fed Weekly Ldlr-ASO	n = 5	n = 5
HFHS-fed Weekly Ldlr-ASO	n = 13	n = 10
Method 2: Genetic strategy		
HFHS-fed *Ldlr*^−/−^	n = 12	n = 15

### Gene and protein expression analyses

For tissue analyses, mice were fasted for 4 h prior to euthanasia. Liver tissue was harvested and snap frozen at −80°C until further analysis. For gene expression analysis, total RNA was extracted from ∼100 mg of whole liver tissue using a commercially available RNA extraction kit according to the manufacturer's protocol (Qiagen RNeasy Mini Kit). After spectroscopic quantification, 2 μg of RNA was reverse-transcribed, and the complementary DNA thus obtained was analyzed by real-time quantitative PCR by standard protocols using an ABI 7900HT instrument. Primer and probe sets for individual genes were purchased from Applied Biosystems (Assay-on-Demand; Life Technologies, Carlsbad, CA; Table 2). *Gapdh* was used as a housekeeping gene, levels of which did not change with the various treatments or genotypes. Relative amounts of the target gene were calculated using the ΔΔCt formula and expressed as a fold change from saline-treated mice. For immunoblot analysis of liver, tissue was homogenized in cold RIPA buffer supplemented with protease inhibitors and centrifuged. Protein concentration was determined using the BCA Protein Assay (Thermo Fisher scientific, Rockford, IL). Immunoblots were performed on equal amounts of protein and probed for the LDLR (catalog no.: AF2255; R&D Systems, goat polyclonal, Minneapolis, MN), ApoB100 and ApoB48 (catalog no.: ab20737; Abcam, rabbit polyclonal, Cambridge, MA), and albumin (catalog no.: AF3329; R&D Systems, goat polyclonal, Minneapolis, MN). Densitometry was performed using ImageJ software (NIH, Bethesda, MD).

**Table 2:** Taqman probe accession numbers

Transcript	Accession Number	Vendor or Source
*Cd68*	Mm03047343_m1	Thermo Fisher Scientific
*Gapdh*	Mm99999915_g1	Thermo Fisher Scientific
*Il6*	Mm00446190_m1	Thermo Fisher Scientific
*Ldlr*	Mm01177349_m1	Thermo Fisher Scientific
*Lgals3*	Mm00802901_m1	Thermo Fisher Scientific
*Saa1*	Mm00656927_g1	Thermo Fisher Scientific
*Saa2*	Mm04208126_mH	Thermo Fisher Scientific
*Saa3*	Mm00441203_m1	Thermo Fisher Scientific

### Plasma analyses

Blood was collected from 4 h fasted mice for plasma isolation. Triglycerides and cholesterol were measured from fasting plasma using colorimetric assays from Fisher Scientific and Roche Diagnostics, as described previously (15). Pooled plasma samples at various time points were subjected to fast-phase liquid chromatography (FPLC), and cholesterol was similarly quantified from collected fractions as previously described (15, 16). Serum amyloid A (Saa) was quantified from plasma using ELISA (17) and likely depicts serum amyloid A1 and serum amyloid A2 because serum amyloid A3 does not circulate in similar models (18). All mice were utilized for all plasma measurements, with the exception of the FPLC time course, in which only a subset of animals were pooled.

### Liver histology and immunohistochemistry

Formalin-fixed paraffin-embedded liver tissue sections were stained with H&E. Histological steatosis and inflammation were assessed semiquantitatively with the scoring system of Kleiner *et al.* (19) by an expert hepatopathologist (Y. L.) in a blinded manner. Since the degree of fibrosis is negative or minimal on H&E-stained sections, a trichrome stain was not performed for evaluation of fibrosis. Macrophages were detected in liver sections immunohistochemically using a rat monoclonal antibody against Mac2 (1:3,000 dilution; monoclonal galectin-3 antibody from Cedarlane Laboratories, Burlington, NC). Area quantification for Mac2 was performed on digital images of immunostained liver sections using image analysis software (ImageJ, NIH, Bethesda, MD).

### Atherosclerosis

Aortas were perfused with saline, the perivascular adipose tissue surrounding the thoracic aorta was completely removed, and the aorta was excised down to the level of the renal bifurcation. Atherosclerosis from the aortic arch was quantified using the en face method using Sudan IV staining as described previously (20). Quantification of aortic sinus lesion area was performed from paraffin-embedded sections of the heart, with every third section (4 μm thick) stained with a monoclonal Mac2 antibody (1:3,000 dilution; Cedarlane Laboratories, Burlington, NC), as described previously (16, 20), with a polyclonal alpha smooth muscle actin (Sma) antibody (1:100 dilution; AbCam, Waltham, MA) or with Movat's pentachrome using standard protocols. Quantification for total lesion size and necrotic core size was performed blinded on digital images of stained tissue sections using Image Pro Plus analysis software, as previously described (16, 20). Movat’s-stained lesions (3–5 consecutive sections per mouse) were utilized to score atherosclerotic lesions according to previous guidelines issued by the American Heart Association for human lesions (21), with some modifications as follows. Sinus lesions were classified as no lesion (I), lesion II (fatty streaks), lesion III (fatty streaks with a fibrous cap), lesion IV (lesions including a necrotic core that makes up <30% of total lesion area), and lesion V (lesions with necrotic cores, fibrotic tissue (yellow), and abundant extracellular matrix (blue)).

### Statistical analyses

Data were analyzed using Prism 6 software (GraphPad, San Diego, CA) and are represented as means ± standard errors. For monthly plasma analyses, the effect of time, differences between treatment groups, and sex differences were evaluated using two-way ANOVA. Significant time-by-group interactions were followed by within-group and between-group post hoc Tukey tests. One-way ANOVA was used to compare differences between treatments for RT-PCR quantification and atherosclerosis. Non-normally distributed variables (i.e., plasma Saa) were log-transformed for analysis and back-transformed for presentation. A *P* value <0.05 was considered statistically significant.

## Results

### Efficacy of pharmacologic and genetic strategies at blunting Ldlr expression

**Fig. 2:**
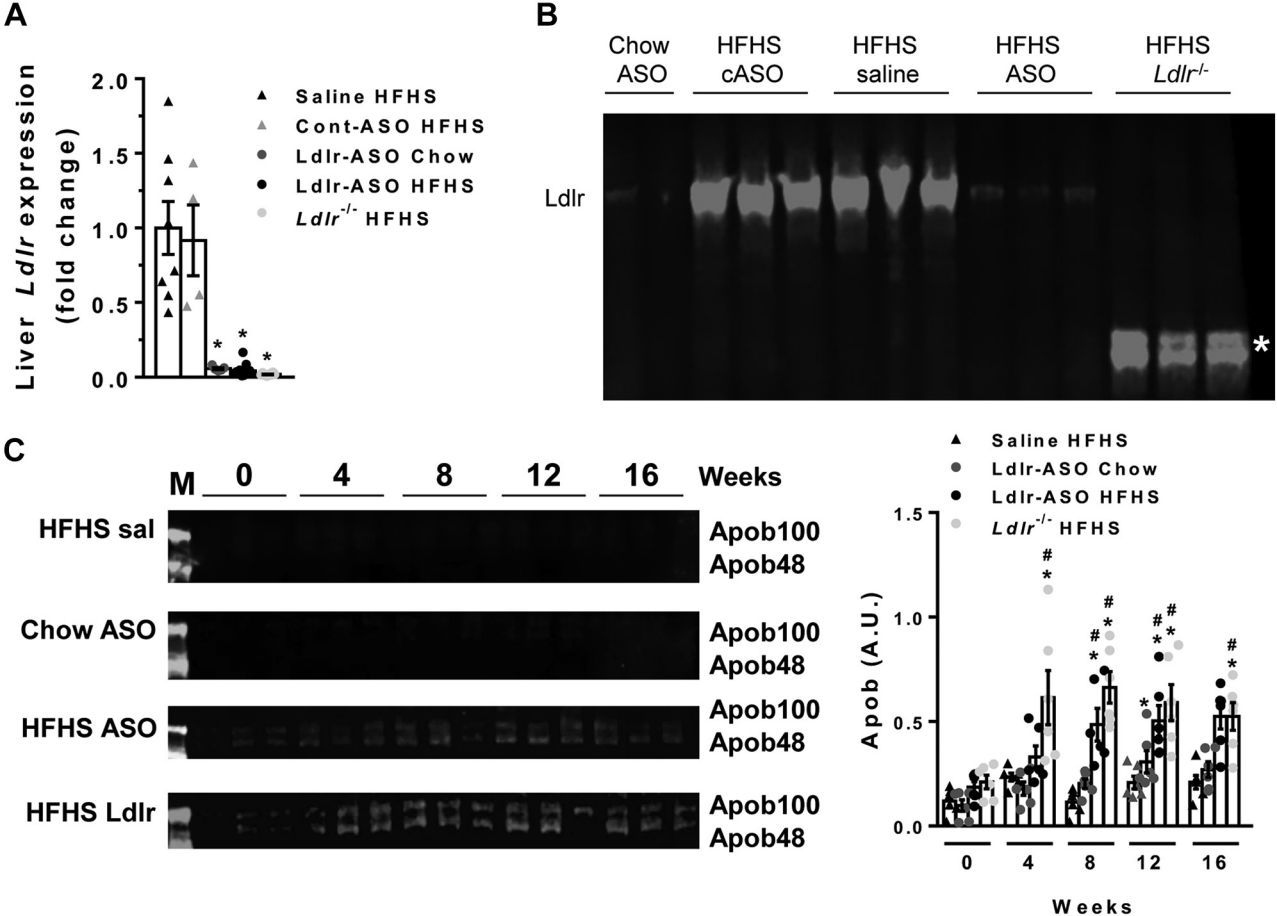
Mouse model validation. A, B: Liver *Ldlr* gene (A, n = 4–16 mice/group) and protein (B, n = 2–3 mice/group) expression levels in the indicated male mice after 16 weeks of chow or HFHS feeding. In (B), the mice genetically deficient in Ldlr show an Ldlr truncation fragment that is not functional (asterisk). C: Apob48 and Apob100 levels in plasma from male mice given once weekly Ldlr-ASO or saline injections for the indicated times (n = 3 mice/group in duplicate) (M = marker). Densitometry for total Apob, normalized to albumin. ∗*P* < 0.05 from saline; #*P* < 0.0 from time 0; and $*P* < 0.05 from HFHS ASO.

Two strategies, one pharmacologic and the other genetic, were employed to promote hyperlipidemia and atherosclerosis in mice. To validate that both strategies blunt Ldlr expression, we measured hepatic Ldlr gene expression from mice given saline, control-ASO, or Ldlr-ASO injections, as well as mice with genetic Ldlr deficiency. Both Ldlr-depleting strategies exhibited comparable reductions in Ldlr gene and protein expression in the liver, with abundant hepatic Ldlr expression in saline-treated and control-ASO-treated mice (Fig. 2A, B). The smaller band observed from the livers of *Ldlr*^−/−^ mice represents a truncation that is not functional (1), indicated by the asterisk in Fig. 2B. There were no notable differences in Ldlr expression levels between males and females in either model of Ldlr deficiency, thus only data from males are shown. We next measured Apob levels in fasted plasma over time beginning at 10 weeks of age (baseline, time = 0 weeks) and every 4 weeks thereafter to confirm that Ldlr-ASO treatment decreases the clearance of Apob100-containing lipoproteins. Neither the HFHS diet alone nor the Ldlr-ASO given to chow-fed mice was sufficient to promote retention of Apob48 or Apob100 in the plasma in male mice (Fig. 2C). However, both Ldlr-ASO and Ldlr genetic deficiency with HFHS diet feeding led to increased Apob levels in the plasma. Thus, both the pharmacological and genetic models of Ldlr deficiency promote comparable levels of Ldlr deficiency when given an HFHS diet, with similar increases in circulating Apob levels.

### Hypercholesterolemia is exacerbated by genetic Ldlr deficiency to a greater extent than Ldlr-ASO

**Fig. 3:**
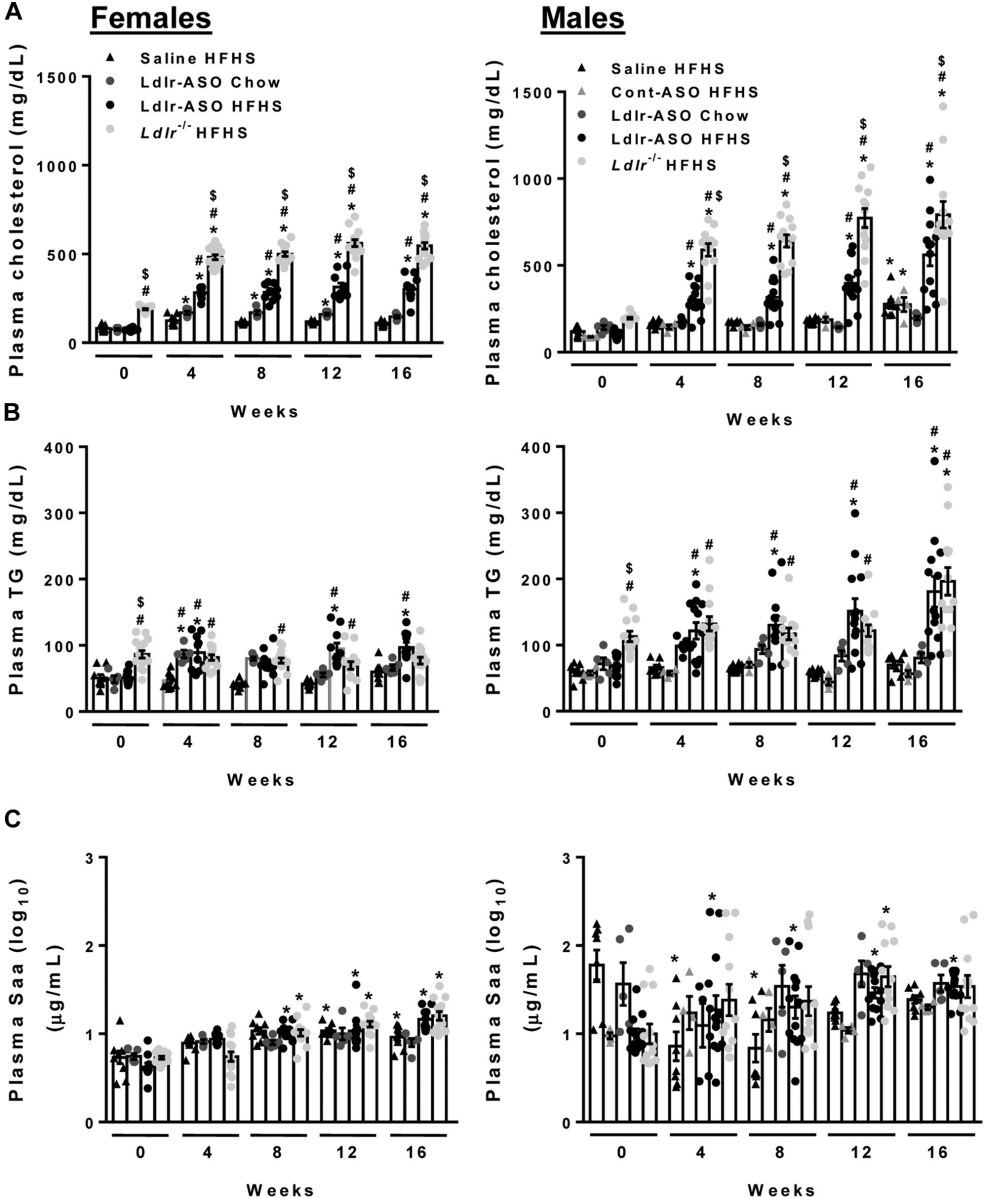
Plasma cholesterol triglyceride and Saa. Plasma was collected from C57Bl6/J male and female mice fed a chow or HFHS diet for 0, 4, 8, 12, and 16 weeks (n = 5–15 mice/group). Mice received weekly saline, control-ASO, or Ldlr-ASO injections or were crossed onto an *Ldlr*^−/−^ background. A: Plasma cholesterol levels. B: Plasma triglyceride (TG) levels. C: Plasma Saa levels. ∗*P* < 0.05 from baseline (time 0), #*P* < 0.05 from saline HFHS, and $*P* < 0.05 from LDLR-ASO.

**Fig. 4:**
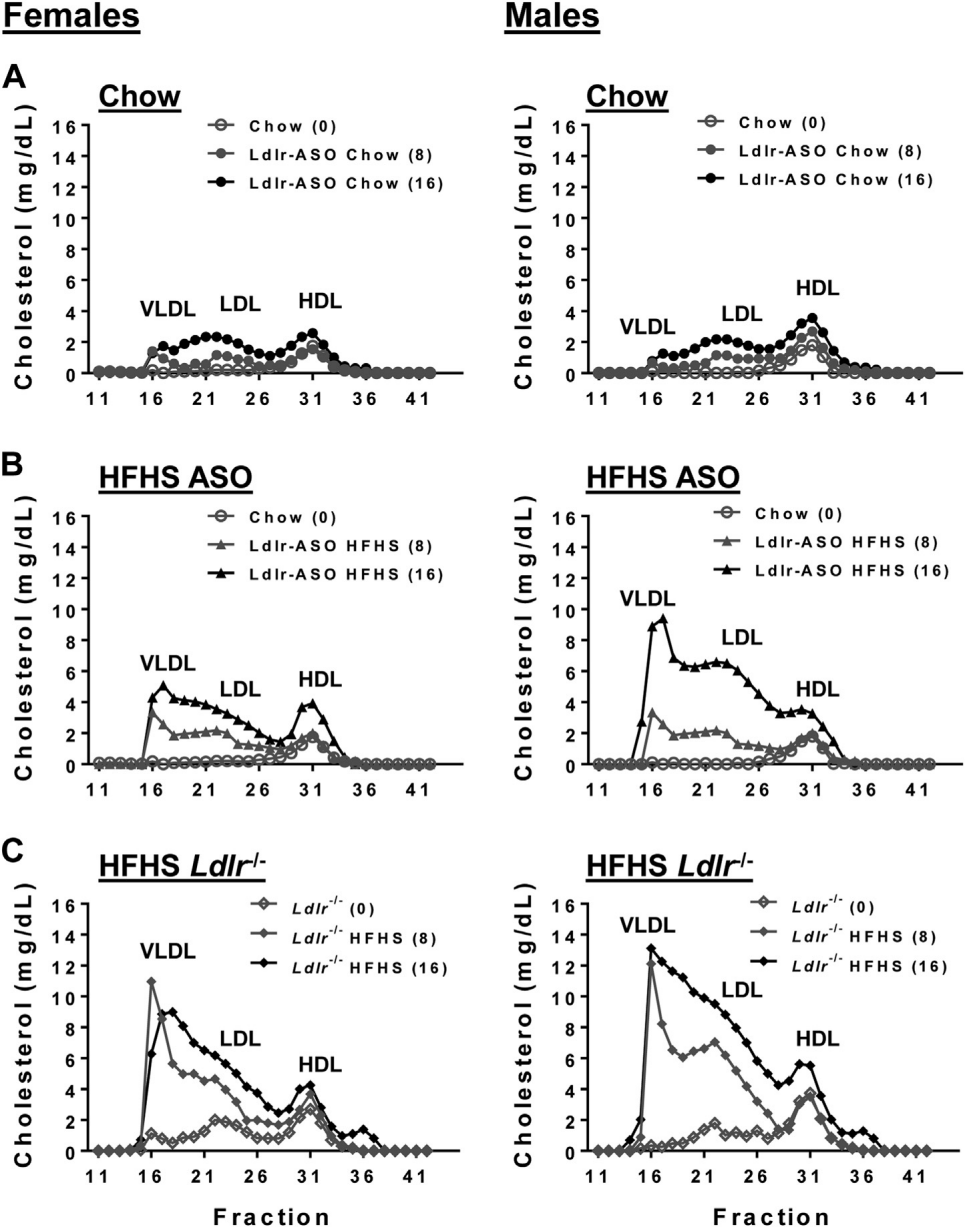
FPLC plasma cholesterol pools over time. Plasma was collected from C57Bl6/J male and female mice fed a chow or HFHS diet for 0, 8, or 16 weeks. Mice received weekly Ldlr-ASO injections or were crossed onto an *Ldlr*^−/−^ background. Plasma from mice was pooled for each treatment group, fractionated using FPLC, and total cholesterol quantified from each fraction. Chow ASO groups (A): n = 5 pooled; HFHS ASO groups (B): n = 9 pooled; and HFHS *Ldlr*^−/−^ groups (C): n = 13.

We next measured fasting plasma cholesterol, triglycerides, and Saa from these mice. In female mice, Ldlr-ASO or *Ldlr*^−/−^ was required to achieve elevated cholesterol levels, regardless of whether mice were fed an HFHS diet (Fig. 3A). Conversely, male mice required the HFHS diet, as no hypercholesterolemia was observed in chow-fed mice given Ldlr-ASO. The combination of HFHS diet with Ldlr-ASO yielded a robust increase in cholesterol levels, an effect that was more pronounced in *Ldlr*^−/−^ mice of both sexes. This is presumably because of a “head start” in this model, as *Ldlr*^−/−^ mice of both sexes exhibited elevated cholesterol levels at baseline (time = 0 weeks). Male mice exhibited significantly higher cholesterol levels than females over time in the Ldlr-deficient cohorts, assessed by two-way ANOVA (Table 3). Both Ldlr-ASO and *Ldlr*^−/−^ comparably increased plasma triglyceride levels, an effect that was more prominent in males (Fig. 3B and Table 3). Plasma Saa levels tended to increase over time in most treatment groups, likely an effect of aging or inflammation induced by hypercholesterolemia, and tended to increase more in male mice deficient in Ldlr (Fig. 3C and Table 3). FPLC plasma fractionation revealed robust differences between the Ldlr depletion models. While plasma cholesterol levels in VLDL, LDL, and HDL increased over time in all groups (Fig. 4), Ldlr-ASO promoted slight increases in cholesterol levels in chow-fed mice, an effect that was much more pronounced in HFHS-fed mice (Fig. 4A, B). Genetic Ldlr deficiency achieved much higher levels of cholesterol in VLDL-containing, LDL-containing, and HDL-containing fractions than in Ldlr-ASO-treated animals (Fig. 4C). Male mice tended to have higher cholesterol levels in FPLC fractions than females. Collectively, these data indicate that hypercholesterolemia induced by genetic Ldlr deficiency exceeded levels achieved pharmacologically.

**Table 3:** Sex differences in plasma lipids, Saa, and Il6

Plasma analyte	Weeks				
	0	4	8	12	16
Plasma cholesterol	*P*	*P*	*P*	*P*	*P*
Saline HFHS	NS	NS	NSN	NS	∗
Ldlr-ASO chow	NS	NS	NS	NS	NS
Ldlr-ASO HFHS	NS	NS	NS	NS	∗∗∗∗
*Ldlr*^−/−^	NS	NS	∗∗	∗∗∗∗	∗∗∗∗
Plasma triglycerides					
Saline HFHS	NS	NS	NS	NS	NS
Ldlr-ASO chow	NS	NS	NS	NS	NS
Ldlr-ASO HFHS	NS	NS	∗∗∗∗	∗∗∗	∗∗∗∗
*Ldlr*^−/−^	NS	∗∗	∗	∗∗	∗∗∗∗
Plasma Saa					
Saline HFHS	∗∗	NS	NS	NS	NS
Ldlr-ASO chow	NS	NS	NS	NS	NS
Ldlr-ASO HFHS	NS	∗	NS	NS	NS
*Ldlr*^−/−^	NS	∗∗	∗	∗	NS
Plasma Il6					
Saline HFHS	N/A	N/A	N/A	N/A	NS
Ldlr-ASO Chow	N/A	N/A	N/A	N/A	NS
Ldlr-ASO HFHS	N/A	N/A	N/A	N/A	∗
*Ldlr*^−/−^	N/A	N/A	N/A	N/A	∗

N/A, not applicable; NS, not significant.

Males versus females, two-way ANOVA with Tukey’s test for multiple comparisons. ∗*P* < 0.05; ∗∗*P* < 0.01; ∗∗∗*P* < 0.001; and ∗∗∗∗*P* < 0.0001.

### Hepatic inflammation is exacerbated by Ldlr-ASO treatment

**Fig. 5:**
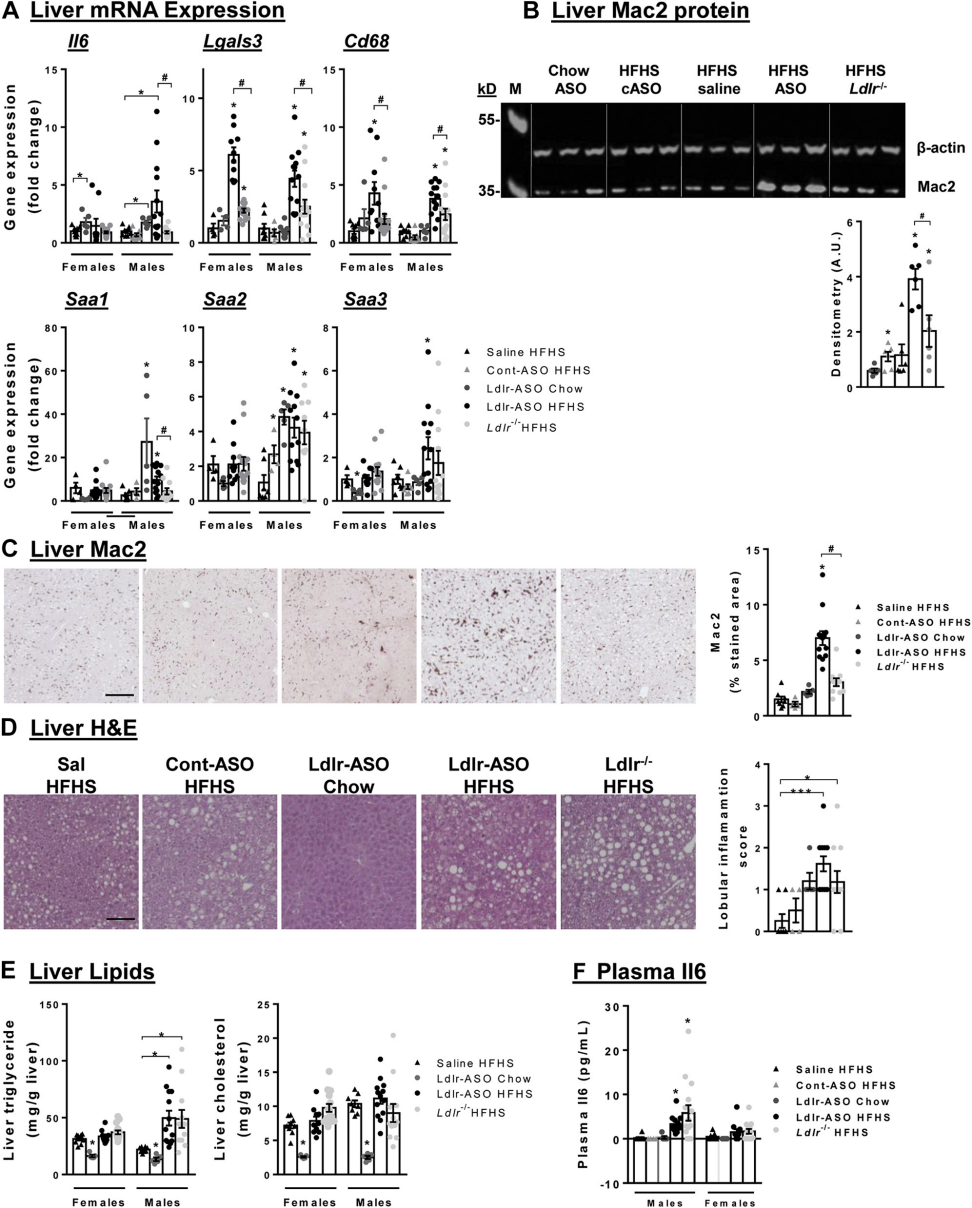
Liver inflammatory gene expression, triglyceride, and cholesterol. Livers were removed from male and female mice that had been injected once weekly with either saline, Ldlr-ASO, or nothing, with or without Ldlr deficiency. A: Expression of inflammatory genes *Il6*, *Lgals3*, *Cd68*, *Saa1*, *Saa2*, and *Saa3* was assessed. B: Representative immunoblot for Mac2 protein, quantified and normalized to β-actin levels (representative of n = 5–6 mice) (M = marker; top band = 55 kD; and bottom band = 35 kD). C, D: Liver histology from Mac2-stained (C) and H&E-stained sections (D), with Mac2 quantification and assessment of lobular inflammation. The scale bar represents 100 μm. E: Hepatic triglycerides and cholesterol were extracted and quantified. F: Plasma Il6 was measured by ELISA. Data are presented as mean ± SEM, n = 5–15 mice/group. ∗*P* < 0.05 from saline; #*P* < 0.05 from Ldlr-ASO.

Because Ldlr-ASO specifically represses Ldlr expression from the liver, we next assessed hepatic inflammation in both models of hypercholesterolemia. Ldlr-ASO delivery to chow-fed mice led to increased expression levels of the cytokines interleukin-6 (*Il6*), serum amyloid A1, and serum amyloid A2 (Fig. 5A). The combination of the HFHS diet with administration of Ldlr-ASO yielded further increases in *Il6* expression in male mice (Fig. 5A). Male and female mice fed the HFHS diet in combination with Ldlr-ASO exhibited robust elevations in the hepatic expression of genes indicative of macrophages, including galectin-3 (*Lgals3*, which encodes or the macrophage marker Mac2) and *Cd68*, with increased expression of the macrophage chemotactic factor serum amyloid A3 observed in males (Fig. 5A). Genetic deficiency of Ldlr did not increase expression of any inflammatory or chemotactic genes, suggesting a specific effect of ASO and not hypercholesterolemia per se. However, *Lgals3* and *Cd68* were modestly increased by genetic Ldlr deficiency, suggesting an increase in hepatic macrophage infiltration. Importantly, administration of control-ASO did not induce measurable inflammatory gene expression, suggesting a specific effect of Ldlr-ASO. Mac2 protein expression was also increased by Ldlr-ASO in HFHS-fed mice, assessed by immunoblot (Fig. 5B) and with immunohistochemistry (Fig. 5C). Lobular inflammatory scoring of H&E-stained liver sections revealed increased hepatic inflammation in both Ldlr-deficient models (Fig. 5D). The administration of Ldlr-ASO did not alter hepatic triglyceride or cholesterol levels (Fig. 5E) or lipid metabolism genes, including carnitine palmitoyl transferase 1 alpha (*Cpt1a*), sterol regulatory element-binding transcription factor 1 (*Srebp1c*), 3-hydroxy-3-methylglutaryl-CoA reductase (*Hmgcr*), diacylglycerol O-acyltransferase 2 (*Dgat2*), and *Fas* (not shown). Plasma Il6 levels were significantly increased in both models of Ldlr deficiency in males but not in females (Fig. 5F). Thus, the administration of Ldlr-ASO appears to promote localized hepatic inflammation beyond what is induced by genetic Ldlr deficiency.

### Genetic Ldlr deficiency promotes more atherosclerosis than pharmacological inhibition of Ldlr

**Fig. 6:**
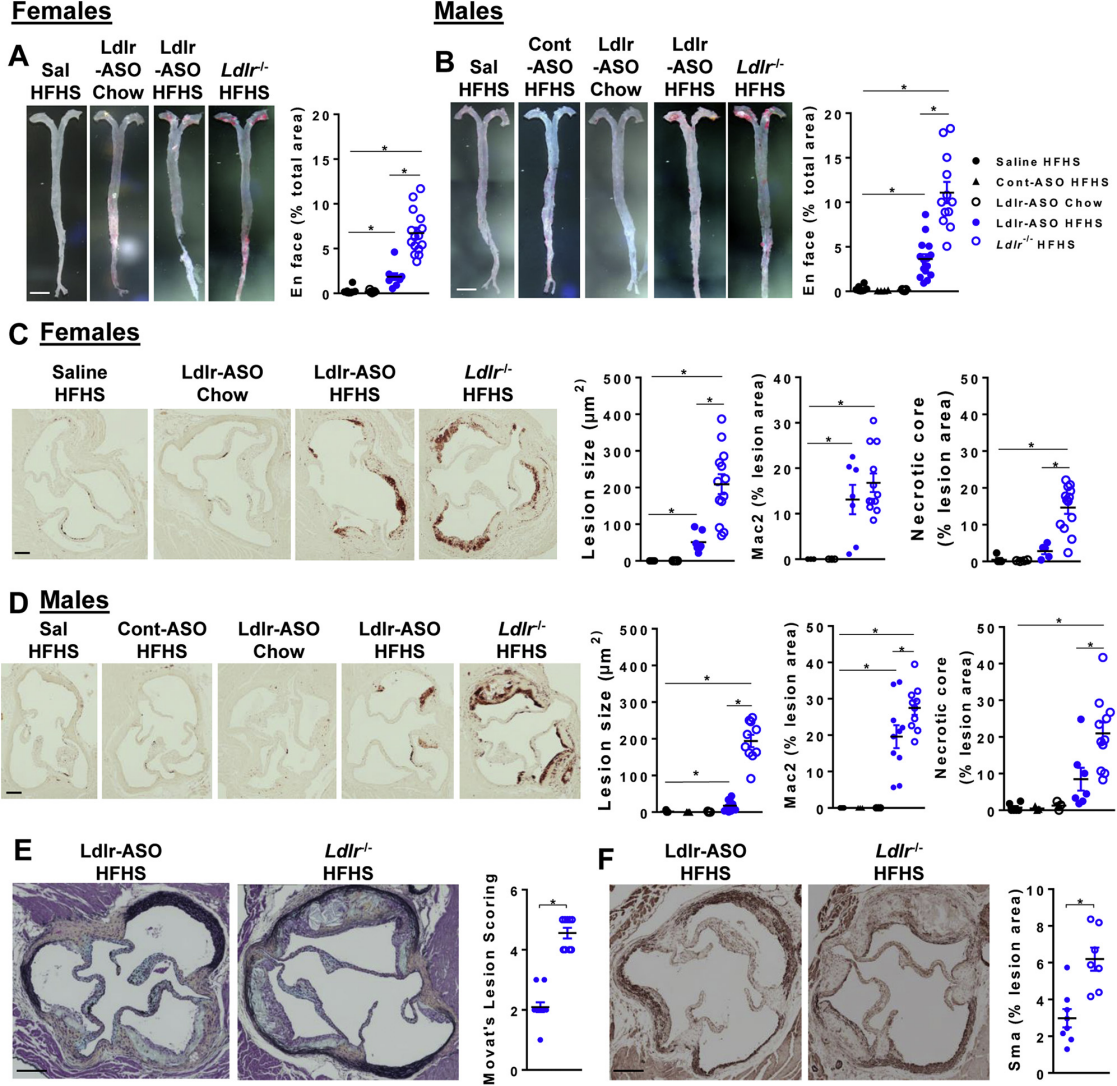
Quantification of atherosclerosis. Aortas and hearts were collected after 16 weeks of the indicated diets and treatments. A, B: Representative images of aortas prepared en face and stained with Sudan IV for females (A) and males (B). The scale bar represents 500 μm. Atherosclerotic area was calculated using ImageJ software and presented as a percentage of the total aortic area. C–F: Representative images of aortic sinuses stained with a Mac-2 antibody from females (C) and males (D) (the scale bar represents 100 μm), Movat’s pentachrome stain for visualization of histology (males, E), and smooth muscle α-actin (males, F) (the scale bar represents 100 μm). Lesion scoring (0–5) was performed as described in the Materials and methods section. Atherosclerotic area, Mac2 staining, necrotic core size, and smooth muscle actin staining were calculated using Image Pro Plus and ImageJ software and presented as a percentage of total lesion size, mean ± SEM. ∗*P* < 0.05 from saline HFHS.

Next, we quantified and characterized atherosclerosis from mice with either pharmacological or genetic inhibition of Ldlr. While Ldlr-ASO promoted modest levels of atherosclerosis in the aorta, *Ldlr*^−/−^ mice had lesions that were 3.6-fold and 3.1-fold greater in size in female and male mice, respectively, consistent with their elevated cholesterol levels (Fig. 6A, B). Male mice exhibited more en face atherosclerosis than female mice (Tables 3 and 4), again consistent with higher cholesterol levels in males. Examination of the aortic sinus revealed similar trends among treatment groups but equivalent lesion size between males and females (Table 4). Utilizing Movat’s pentachrome stain, we found that male mice given Ldlr-ASO developed small lesions that consisted of fatty streaks with an occasional thin fibrous cap (Fig. 6E). Conversely, male mice with genetic Ldlr deficiency developed lesions that were 4-fold and 11-fold larger and more advanced, with evidence of larger necrotic cores, extracellular matrix (blue), and fibrotic tissue (yellow) (Fig. 6E). Staining for the pan-macrophage marker, Mac2, revealed that the aortic sinus was equivalently enriched in macrophages from female mice in both Ldlr-deficient models, while aortic sinus lesions from *Ldlr*^−/−^ males exhibited higher Mac2 immunostaining than those with pharmacological Ldlr disruption (Fig. 6E, F). Smooth muscle actin (Sma) staining was 2-fold greater in *Ldlr*^−/−^ mice than in those given Ldlr-ASO (Fig. 6F). Both male and female *Ldlr*^−/−^ mice had larger necrotic cores as a percentage of total lesion area, and more fibrosis as indicated by more abundant yellow in the H&E stains, suggesting that genetic Ldlr deficiency leads to more advanced lesions. Collectively, genetic deficiency of Ldlr promotes the formation of larger more advanced atherosclerotic lesions in both male and female mice.

**Table 4:** Sex differences in atherosclerosis

Treatment group	En Face Atherosclerosis	Aortic Sinus Atherosclerosis	Mac2 Staining	Necrotic Core Area
Saline HFHS	NS	NS	NS	NS
Ldlr-ASO chow	NS	NS	NS	NS
Ldlr-ASO HFHS	NS	NS	NS	NS
*Ldlr*^−/−^	∗∗∗∗	NS	∗	NS

NS, not significant.

Males versus females, two-way ANOVA with Tukey’s test for multiple comparisons. ∗*P* < 0.05; ∗∗∗∗*P* < 0.0001.

## Discussion

In this study, we show that 16 weeks of Ldlr-ASO treatment in conjunction with an HFHS diet can effectively increase plasma cholesterol levels sufficiently to promote atherosclerosis in male and female mice. However, genetic Ldlr deficiency in age-matched and sex-matched mice yielded larger lesions, larger necrotic cores, higher macrophage content, higher Movat’s lesion scoring, and more Sma staining, indicative of more advanced lesions. The method of Ldlr-ASO administration used in this study, including dosage and timing of delivery, is the same as has been used in previous studies (9, 10, 11, 22). Our results on plasma cholesterol and atherosclerosis levels are in agreement with most of these studies, which used a similar Western-type rodent diet, although with slightly higher cholesterol content (9, 10). While Pennig *et al.* (22) achieved higher levels of plasma cholesterol (800 vs. 600 mg/dl) and aortic root atherosclerotic area (3 × 10^5^ μm^2^ vs. 0.5 × 10^5^ μm^2^) than in our study, mice in that study were fed a diet containing eight times more cholesterol. Thus, the dietary conditions utilized could be an important determinant of the efficacy of Ldlr-ASO compared with genetic Ldlr deficiency.

It also is possible that *Ldlr*^−/−^ mice were given a “head start” by virtue of being Ldlr deficient since birth, whereas mice given Ldlr-ASO began Ldlr deficiency 10 weeks later. It has been suggested previously that mice treated with Ldlr-ASO could develop less atherosclerosis than *Ldlr*^−/−^ mice because Ldlr-ASO, which strongly targets the liver, is unlikely to reduce Ldlr expression in other Ldlr-expressing cells such as macrophages (9), thereby impacting lesion development (23, 24). Another potential reason could be related to the study design. Ldlr-ASO was administered once weekly to mice, and blood was collected for analyses just prior to weekly injections, potentially enabling Ldlr levels to have transiently risen by then. A potential “yo-yo” effect on dampening Ldlr expression could explain the consistently observed less advanced atherosclerosis in mice given Ldlr-ASO and represents a caveat to our study design. Future studies could examine Ldlr expression or plasma cholesterol kinetics with more frequent sampling to determine the extent of weekly cholesterol fluctuations, should any exist.

It is well known that atherosclerosis is a disease process heavily influenced by both hyperlipidemia and inflammation. Lipoproteins such as LDL and remnants of triglyceride-rich lipoproteins are retained in the artery wall, become modified (e.g., oxidized), and evoke a systemic inflammatory response that further perpetuates atherosclerotic lesion development (25, 26). Of our two models, *Ldlr*^−/−^ mice displayed higher cholesterol levels and larger more advanced atherosclerotic lesions than mice given Ldlr-ASO, despite the increased levels of hepatic inflammation observed in Ldlr-ASO-treated mice and comparable plasma Saa and Il6 levels and lesion macrophage content from both models. This suggests that atherosclerosis is heavily influenced by hypercholesterolemia in these models. Moreover, male mice consistently displayed higher plasma cholesterol levels than female mice, which translated into larger and more advanced atherosclerotic lesions. Male and female mice showed comparable hepatic inflammation and plasma Saa levels, again pointing toward hypercholesterolemia as an important driver of atherosclerosis in these models. However, male mice also displayed higher proportions of lesion macrophages than females in both Ldlr-deficiency models, suggesting a potential contribution from local inflammation; however, the inherent phenotypes of these lesion macrophages are unknown. Thus, it cannot be conclusively determined whether elevated plasma cholesterol or lesion macrophage content was more influential in the increased atherosclerotic development in male *Ldlr*^−/−^ mice.

Our observation that Ldlr-ASO exacerbates HFHS diet-mediated hepatic inflammation raises some concerns for its use, especially for promoting a disease state that so closely involves inflammation. Some first-generation ASOs exhibited proinflammatory effects (27), which led to subsequent changes in development for successive ASO generations. Previous studies have suggested that ASOs have inherent immunostimulatory potential in cultured human peripheral blood mononuclear cells (28), mice (29, 30), and nonhuman primates (31, 32). Similarly, some ASOs have been reported to induce mild to moderate levels of hepatic toxicity in rats, nonhuman primates, and humans (33). As such, the chemical composition of ASOs is constantly evolving to improve target specificity while minimizing off-target effects, toxicity, and inflammation. Importantly, the Ldlr-ASO used in the present study was the GalNAc version, enabling Ldlr inhibition at lower doses with the potential for lower toxicity.

While not a main objective in this work, we also describe significant differences between male and female mice in these models. In humans, men are at a higher risk for the development of cardiovascular disease than women, an effect that wanes with aging and the onset of menopause in women (34, 35). By contrast, it has previously been suggested that female *Ldlr*^−/−^ mice develop larger and more advanced atherosclerotic lesions than age-matched male mice (36). However, our laboratory has previously shown elevated atherosclerotic burden in male mice (16), an effect also observed in the current study. The reasons for this are not immediately clear but could simply be reflective of the nuanced details in our study design. It has been reported that age plays a major role in the sex differences observed between male and female mice, with male and female mice older than 6 months of age displaying equivalent levels of atherosclerosis (36). Because our mice are 26 weeks at study termination, this could explain why males exhibit elevated atherosclerosis levels in our studies.

In summary, we present evidence suggesting that while Ldlr-ASO is an acceptable strategy to increase Apob-containing lipoproteins for subsequent atherosclerosis development when mice are fed a high fat and cholesterol-containing diet, higher cholesterol levels and more advanced lesions are achieved in age-matched *Ldlr*^−/−^ mice. However, these results do not minimize the validity of Ldlr-ASO as a critical tool to study atherosclerosis regression or as a useful tool in complex genetic models of atherosclerosis. One might envision situations where it is desirable to study early atherosclerotic lesions, such as testing anti-inflammatory compounds or lipid-lowering remedies. Regardless, care should be taken to implement the optimal study design for Ldlr-ASO use, noting the potential for hepatic inflammation in Ldlr-ASO models.

## Data availability

All data pertaining to this study are contained within the article.

## Conflict of interest

The authors declare that they have no conflicts of interest with the contents of this article.

## References

[1] Ishibashi S., Brown M.S., Goldstein J.L., Gerard R.D., Hammer R.E., Herz J. (1993). Hypercholesterolemia in low density lipoprotein receptor knockout mice and its reversal by adenovirus-mediated gene delivery. J. Clin. Invest..

[2] Zhang S.H., Reddick R.L., Piedrahita J.A., Maeda N. (1992). Spontaneous hypercholesterolemia and arterial lesions in mice lacking apolipoprotein E. Science.

[3] Bennett C.F., Swayze E.E. (2010). RNA targeting therapeutics: molecular mechanisms of antisense oligonucleotides as a therapeutic platform. Annu. Rev. Pharmacol. Toxicol..

[4] Tromp T.R., Stroes E.S.G., Hovingh G.K. (2020). Gene-based therapy in lipid management: the winding road from promise to practice. Expert Opin. Investig. Drugs.

[5] Kastelein J.J., Wedel M.K., Baker B.F., Su J., Bradley J.D., Yu R.Z., Chuang E., Graham M.J., Crooke R.M. (2006). Potent reduction of apolipoprotein B and low-density lipoprotein cholesterol by short-term administration of an antisense inhibitor of apolipoprotein B. Circulation.

[6] Alexander V.J., Xia S., Hurh E., Hughes S.G., O'Dea L., Geary R.S., Witztum J.L., Tsimikas S. (2019). N-acetyl galactosamine-conjugated antisense drug to APOC3 mRNA, triglycerides and atherogenic lipoprotein levels. Eur. Heart J..

[7] Graham M.J., Lee R.G., Brandt T.A., Tai L.J., Fu W., Peralta R., Yu R., Hurh E., Paz E., McEvoy B.W., Baker B.F., Pham N.C., Digenio A., Hughes S.G., Geary R.S. (2017). Cardiovascular and metabolic effects of ANGPTL3 antisense oligonucleotides. N. Engl. J. Med..

[8] Tsimikas S., Karwatowska-Prokopczuk E., Gouni-Berthold I., Tardif J.C., Baum S.J., Steinhagen-Thiessen E., Shapiro M.D., Stroes E.S., Moriarty P.M., Nordestgaard B.G., Xia S., Guerriero J., Viney N.J., O'Dea L., Witztum J.L. (2020). Lipoprotein(a) reduction in persons with cardiovascular disease. N. Engl. J. Med..

[9] Basu D., Hu Y., Huggins L.A., Mullick A.E., Graham M.J., Wietecha T., Barnhart S., Mogul A., Pfeiffer K., Zirlik A., Fisher E.A., Bornfeldt K.E., Willecke F., Goldberg I.J. (2018). Novel reversible model of atherosclerosis and regression using oligonucleotide regulation of the LDL receptor. Circ. Res..

[10] Josefs T., Basu D., Vaisar T., Arets B., Kanter J.E., Huggins L.A., Hu Y., Liu J., Clouet-Foraison N., Heinecke J.W., Bornfeldt K.E., Goldberg I.J., Fisher E.A. (2021). Atherosclerosis regression and cholesterol efflux in hypertriglyceridemic mice. Circ. Res..

[11] Bashore A.C., Liu M., Key C.C., Boudyguina E., Wang X., Carroll C.M., Sawyer J.K., Mullick A.E., Lee R.G., Macauley S.L., Parks J.S. (2019). Targeted deletion of hepatocyte Abca1 increases plasma HDL (high-density lipoprotein) reverse cholesterol transport via the LDL (low-density lipoprotein) receptor. Arterioscler. Thromb. Vasc. Biol..

[12] Getz G.S., Reardon C.A. (2016). Do the Apoe-/- and Ldlr-/- mice yield the same insight on atherogenesis?. Arterioscler. Thromb. Vasc. Biol..

[13] Subramanian S., Han C., Chiba T., McMillen T., Wang S., Haw A.R., Kirk E., O'Brien K., Chait A. (2008). Dietary cholesterol worsens adipose tissue macrophage accumulation and atherosclerosis in obese LDL receptor-deficient mice. Arterioscler. Thromb. Vasc. Biol..

[14] den Hartigh L.J., Wang S., Goodspeed L., Wietecha T., Houston B., Omer M., Ogimoto K., Subramanian S., Gowda G.A., O'Brien K.D., Kaiyala K.J., Morton G.J., Chait A. (2017). Metabolically distinct weight loss by 10,12 CLA and caloric restriction highlight the importance of subcutaneous white adipose tissue for glucose homeostasis in mice. PLoS One.

[15] Lewis K.E., Kirk E.A., McDonald T.O., Wang S., Wight T.N., O'Brien K.D., Chait A. (2004). Increase in serum amyloid a evoked by dietary cholesterol is associated with increased atherosclerosis in mice. Circulation.

[16] Chait A., Wang S., Goodspeed L., Gomes D., Turk K.E., Wietecha T., Tang J., Storey C., O'Brien K.D., Rubinow K.B., Tang C., Vaisar T., Gharib S.A., Lusis A.J., Den Hartigh L.J. (2021). Sexually dimorphic relationships among Saa3 (serum amyloid A3), inflammation, and cholesterol metabolism modulate atherosclerosis in mice. Arterioscler. Thromb. Vasc. Biol..

[17] den Hartigh L.J., Wang S., Goodspeed L., Ding Y., Averill M., Subramanian S., Wietecha T., O'Brien K.D., Chait A. (2014). Deletion of serum amyloid A3 improves high fat high sucrose diet-induced adipose tissue inflammation and hyperlipidemia in female mice. PLoS One.

[18] Chiba T., Han C.Y., Vaisar T., Shimokado K., Kargi A., Chen M.H., Wang S., McDonald T.O., O'Brien K.D., Heinecke J.W., Chait A. (2009). Serum amyloid A3 does not contribute to circulating SAA levels. J. Lipid Res..

[19] Kleiner D.E., Brunt E.M., Van Natta M., Behling C., Contos M.J., Cummings O.W., Ferrell L.D., Liu Y.C., Torbenson M.S., Unalp-Arida A., Yeh M., McCullough A.J., Sanyal A.J., Nonalcoholic Steatohepatitis Clinical Research Network (2005). Design and validation of a histological scoring system for nonalcoholic fatty liver disease. Hepatology.

[20] Kanter J.E., Goodspeed L., Wang S., Kramer F., Wietecha T., Gomes-Kjerulf D., Subramanian S., O'Brien K.D., den Hartigh L.J. (2018). 10,12 Conjugated linoleic acid-driven weight loss is protective against atherosclerosis in mice and is associated with alternative macrophage enrichment in perivascular adipose tissue. Nutrients.

[21] Stary H.C., Chandler A.B., Dinsmore R.E., Fuster V., Glagov S., Insull W., Rosenfeld M.E., Schwartz C.J., Wagner W.D., Wissler R.W. (1995). A definition of advanced types of atherosclerotic lesions and a histological classification of atherosclerosis. A report from the Committee on Vascular Lesions of the Council on Arteriosclerosis, American Heart Association. Circulation.

[22] Pennig J., Scherrer P., Gissler M.C., Anto-Michel N., Hoppe N., Füner L., Härdtner C., Stachon P., Wolf D., Hilgendorf I., Mullick A., Bode C., Zirlik A., Goldberg I.J., Willecke F. (2019). Glucose lowering by SGLT2-inhibitor empagliflozin accelerates atherosclerosis regression in hyperglycemic STZ-diabetic mice. Sci. Rep..

[23] Herijgers N., Van Eck M., Groot P.H., Hoogerbrugge P.M., Van Berkel T.J. (2000). Low density lipoprotein receptor of macrophages facilitates atherosclerotic lesion formation in C57Bl/6 mice. Arterioscler. Thromb. Vasc. Biol..

[24] Linton M.F., Babaev V.R., Gleaves L.A., Fazio S. (1999). A direct role for the macrophage low density lipoprotein receptor in atherosclerotic lesion formation. J. Biol. Chem..

[25] Tannock L.R., Chait A. (2004). Lipoprotein-matrix interactions in macrovascular disease in diabetes. Front. Biosci..

[26] Ross R. (1999). Atherosclerosis--an inflammatory disease. N. Engl. J. Med..

[27] Frazier K.S. (2015). Antisense oligonucleotide therapies: the promise and the challenges from a toxicologic pathologist's perspective. Toxicol. Pathol..

[28] Hartmann G., Krug A., Waller-Fontaine K., Endres S. (1996). Oligodeoxynucleotides enhance lipopolysaccharide-stimulated synthesis of tumor necrosis factor: dependence on phosphorothioate modification and reversal by heparin. Mol. Med..

[29] Senn J.J., Burel S., Henry S.P. (2005). Non-CpG-containing antisense 2'-methoxyethyl oligonucleotides activate a proinflammatory response independent of Toll-like receptor 9 or myeloid differentiation factor 88. J. Pharmacol. Exp. Ther..

[30] Burel S.A., Han S.R., Lee H.S., Norris D.A., Lee B.S., Machemer T., Park S.Y., Zhou T., He G., Kim Y., MacLeod A.R., Monia B.P., Lio S., Kim T.W., Henry S.P. (2013). Preclinical evaluation of the toxicological effects of a novel constrained ethyl modified antisense compound targeting signal transducer and activator of transcription 3 in mice and cynomolgus monkeys. Nucleic Acid Ther..

[31] Burel S.A., Machemer T., Ragone F.L., Kato H., Cauntay P., Greenlee S., Salim A., Gaarde W.A., Hung G., Peralta R., Freier S.M., Henry S.P. (2012). Unique O-methoxyethyl ribose-DNA chimeric oligonucleotide induces an atypical melanoma differentiation-associated gene 5-dependent induction of type I interferon response. J. Pharmacol. Exp. Ther..

[32] Farman C.A., Kornbrust D.J. (2003). Oligodeoxynucleotide studies in primates: antisense and immune stimulatory indications. Toxicol. Pathol..

[33] Burdick A.D., Sciabola S., Mantena S.R., Hollingshead B.D., Stanton R., Warneke J.A., Zeng M., Martsen E., Medvedev A., Makarov S.S., Reed L.A., Davis J.W., Whiteley L.O. (2014). Sequence motifs associated with hepatotoxicity of locked nucleic acid--modified antisense oligonucleotides. Nucleic Acids Res..

[34] Halvorsen D.S., Johnsen S.H., Mathiesen E.B., Njølstad I. (2009). The association between inflammatory markers and carotid atherosclerosis is sex dependent: the Tromsø study. Cerebrovasc. Dis..

[35] Sinning C., Wild P.S., Echevarria F.M., Wilde S., Schnabel R., Lubos E., Herkenhoff S., Bickel C., Klimpe S., Gori T., Münzel T.F., Blankenberg S., Espinola-Klein C., Gutenberg-Heart Study (2011). Sex differences in early carotid atherosclerosis (from the community-based Gutenberg-Heart Study). Am. J. Cardiol..

[36] Man J.J., Beckman J.A., Jaffe I.Z. (2020). Sex as a biological variable in atherosclerosis. Circ. Res..

